# Sample-based modeling reveals bidirectional interplay between cell cycle progression and extrinsic apoptosis

**DOI:** 10.1371/journal.pcbi.1007812

**Published:** 2020-06-04

**Authors:** Dirke Imig, Nadine Pollak, Frank Allgöwer, Markus Rehm

**Affiliations:** 1 University of Stuttgart, Institute for Systems Theory and Automatic Control, Pfaffenwaldring 9, Stuttgart, Germany; 2 University of Stuttgart, Institute of Cell Biology and Immunology, Allmandring 31, Stuttgart, Germany; 3 University of Stuttgart, Stuttgart Research Center Systems Biology, Nobelstr. 15, Stuttgart, Germany; King’s College London, UNITED KINGDOM

## Abstract

Apoptotic cell death can be initiated through the extrinsic and intrinsic signaling pathways. While cell cycle progression promotes the responsiveness to intrinsic apoptosis induced by genotoxic stress or spindle poisons, this has not yet been studied conclusively for extrinsic apoptosis. Here, we combined fluorescence-based time-lapse monitoring of cell cycle progression and cell death execution by long-term time-lapse microscopy with sampling-based mathematical modeling to study cell cycle dependency of TRAIL-induced extrinsic apoptosis in NCI-H460/geminin cells. In particular, we investigated the interaction of cell death timing and progression of cell cycle states. We not only found that TRAIL prolongs cycle progression, but in reverse also that cell cycle progression affects the kinetics of TRAIL-induced apoptosis: Cells exposed to TRAIL in G_1_ died significantly faster than cells stimulated in S/G_2_/M. The connection between cell cycle state and apoptosis progression was captured by developing a mathematical model, for which parameter estimation revealed that apoptosis progression decelerates in the second half of the cell cycle. Similar results were also obtained when studying HCT-116 cells. Our results therefore reject the null hypothesis of independence between cell cycle progression and extrinsic apoptosis and, supported by simulations and experiments of synchronized cell populations, suggest that unwanted escape from TRAIL-induced apoptosis can be reduced by enriching the fraction of cells in G_1_ phase. Besides novel insight into the interrelation of cell cycle progression and extrinsic apoptosis signaling kinetics, our findings are therefore also relevant for optimizing future TRAIL-based treatment strategies.

## Author summary

TRAIL (TNF-related apoptosis-inducing ligand) induces cell death preferentially in cancer cells. Whether cell cycle progression notably affects extrinsic apoptosis has remained unclear, since systematic experimental and mathematical studies to quantitatively understand such interdependencies remained challenging. Here, we applied statistical and mechanistic modeling, linked with experimental analyses, to determine if cell cycle and apoptosis progression are interconnected. Using sample-based modeling, we demonstrate that times required to commit apoptotic cell death depend on the cell cycle position at the time of TRAIL exposure, with delays manifesting during S phase, around a time that can be defined as a point of apoptosis deceleration (PAD). Overall, cells receiving TRAIL in the G_1_ phase were more likely to die, providing scope to optimize TRAIL-based treatment strategies with respect to cell cycle dynamics.

## Introduction

The cell cycle regulates the expression of numerous proteins [1, 2], and consequently affects various cellular processes, including for example glycolysis and oxidative phosphorylation [3]. Fluorescent, ubiquitination-based cell cycle indicator (Fucci) reporter systems [4] allow distinction between G_1_ and S/G_2_/M phases by coupling fluorescent markers to proteins that accumulate in specific cell cycle phases. As a result, start and end points of Fucci phases can be tracked in parallel to other processes such as cell death of single cells in long-term time-lapse microscopy experiments [5]. A connection between cell cycle progression and apoptosis, for example induced by irradiation or chemotherapeutics, was proposed years ago due to similar morphology and involvement of mutual genes [6]. Long-term time-lapse experiments already provided insight into the cell cycle dependency of intrinsic apoptosis [7–12]. Concerning TRAIL-induced extrinsic apoptosis, the role of the cell cycle instead remains vague and underexplored. Canonical extrinsic apoptosis requires the activation of death receptors, such as TRAIL receptors 1 and 2, which is followed by a signaling cascade that leads to cleavage of executioner caspases (-3, -6 and -7) and subsequent cell death. Starting with binding of TRAIL and cluster formation of death receptors, cell-intrinsic signaling activates caspase-8. Apoptosis is either directly induced by caspase-8-dependent cleavage of pro-caspase-3 (type I) or relies on Bid cleavage and the permeabilization of the outer mitochondrial membrane (type II) [13]. Since *de novo* synthesis of proteins subsequent to TRAIL exposure is not required for apoptosis induction, independence between extrinsic apoptosis and cell cycle progression could be expected. On the other hand, expression, phosphorylation and localization of several proteins involved in signal transduction is controlled in a cell cycle-dependent manner [14–16]. To study if both dynamical processes, extrinsic apoptosis and cell cycle progression, are coupled, and due to substantial cell-to-cell heterogeneities even in isogenic cell populations [12, 17], the development and application of mathematical models and appropriate statistical tools is inevitable. Mathematical modeling of the cell cycle machinery has a long history (e.g. [18, 19]), including studies integrating time-lapse microscopy data of Fucci reporter cells (e.g. [20]), but modeling studies linking extrinsic apoptosis and cell cycle dynamics have not yet been conducted. Where initial work in this direction was attempted, modeling approaches linked proliferation and cell death to defined signaling networks [21, 22]). Although complex models are necessary for the understanding of signal transduction kinetics and the role of cellular heterogeneity and noise in cell populations [23], parametrization of high-resolution signaling models requires a significant amount of data and biological knowledge. A preceding step for describing and quantifying possible interconnections between cell cycle progression and extrinsic apoptosis signaling is defining parameters phenomenologically. Here, we therefore focused on statistical methods and phenomenological models to study the relationship of extrinsic apoptosis and cell cycle progression in NCI-H460/geminin cells [24] and HCT-116/geminin cells when these were exposed to a 2nd generation hexavalent TRAIL receptor agonist (IZI1551) [25].

## Results

### Cells in S/G_2_/M phase require longer to die than cells treated in G_1_ phase

**Fig 1:**
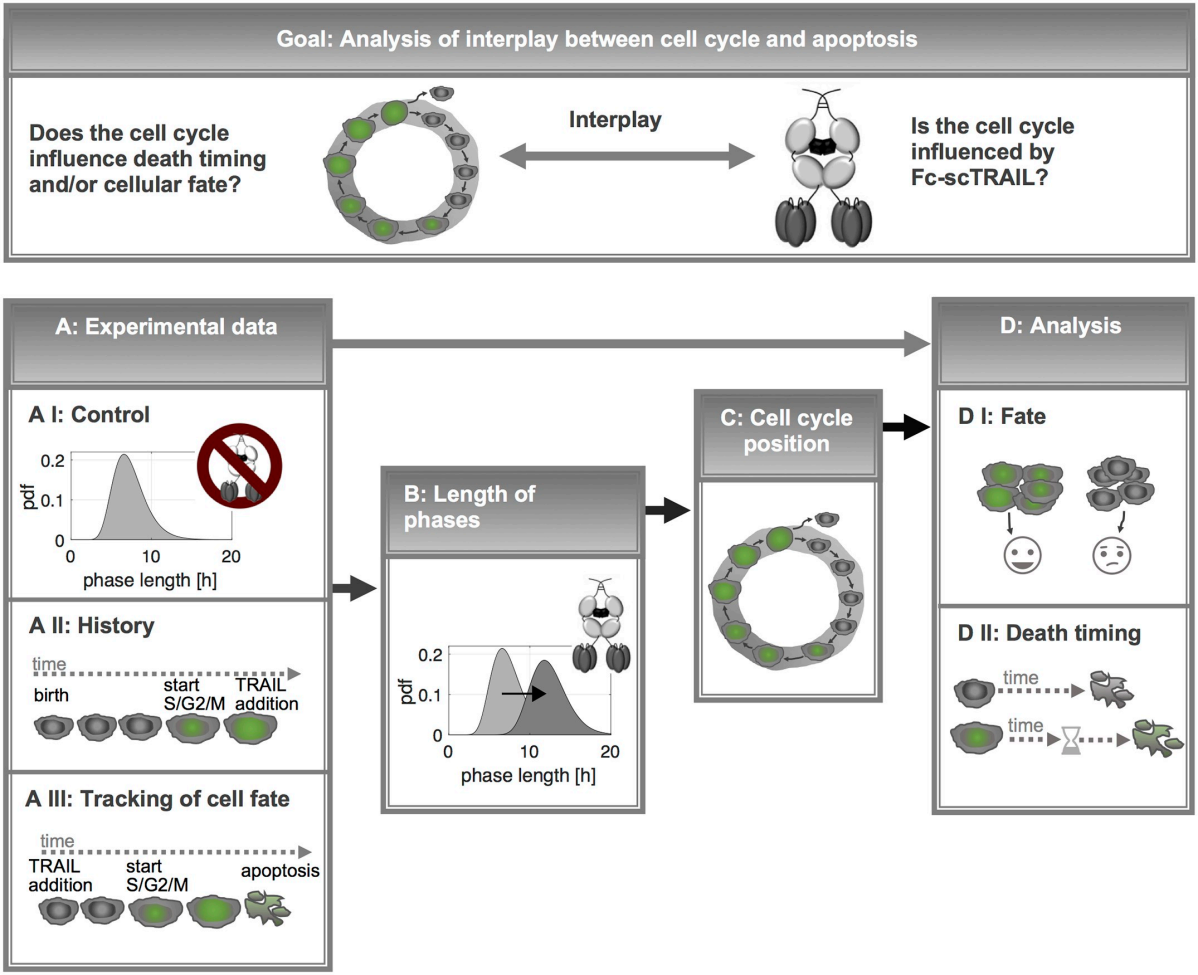
Framework to explore the interdependence of cell death and cell cycle. Arrows indicate information flow from experimental measurements to data normalization and analysis. The two key questions for exploring the apoptosis/cell cycle connection were: Does the cell cycle influence death timing and/or cellular fate? Is the cell cycle influenced by TRAIL? To answer these questions, the following measurements were required: A I) length of phases in untreated cells, A II) the history of cells that were stimulated with TRAIL (end of previous phase), A III) durations from TRAIL exposure to cell death or to the end of a cell cycle phase. On basis of this data, the influence of TRAIL on the phase length was analyzed (B). Information about the latter was incorporated to estimate the initial cell cycle position at TRAIL addition (C). Results were used to analyze the influence of the initial cell cycle position on cell fate and death timing (D).

**Fig 2:**
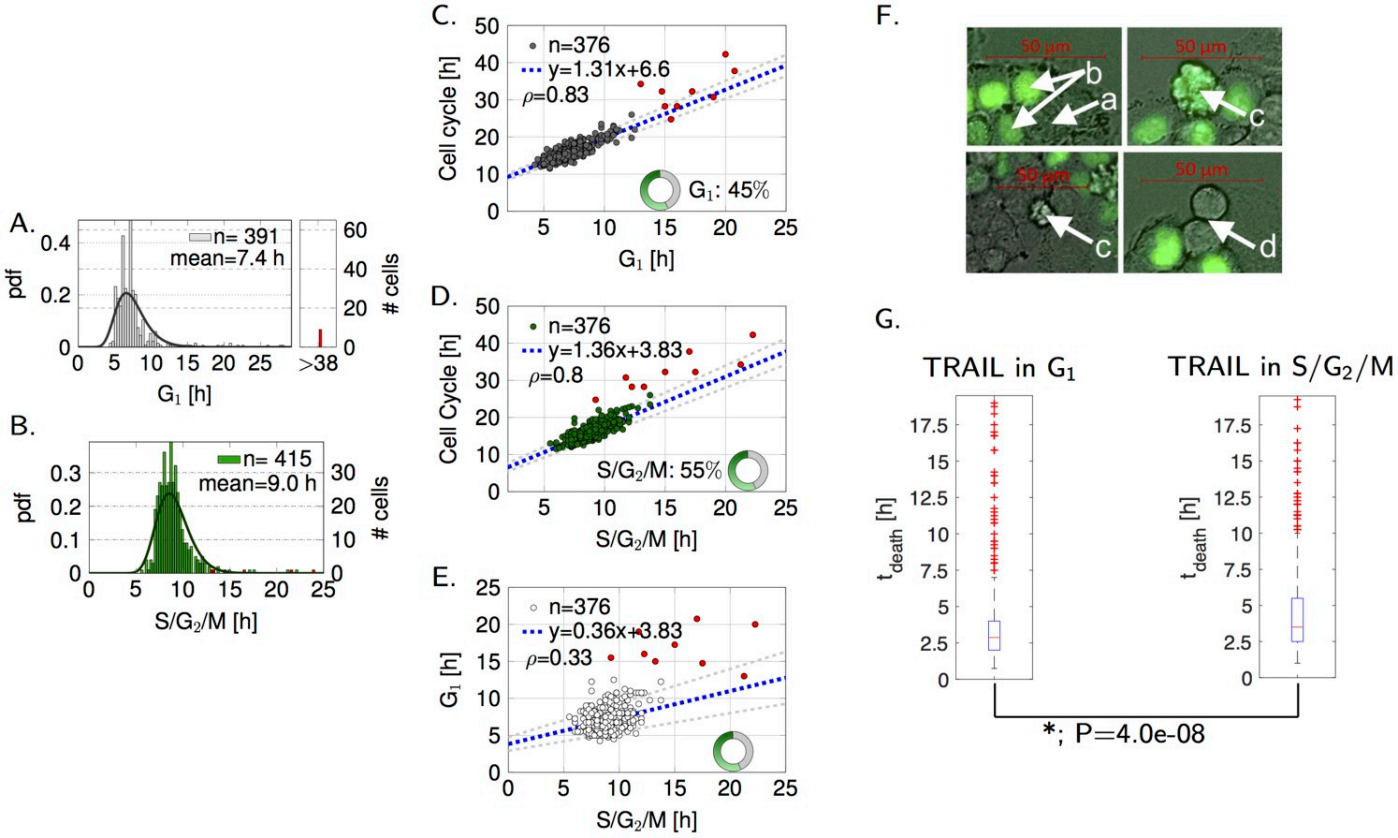
Long-term time-lapse fluorescent microscopy can be employed to characterize cell cycle characteristics of asynchronous cell populations. (A,B) Length of G_1_ and S/G_2_/M phases in control cells. Parameters of the fitted lognormal distributions are *μ*_G1_ = 1.96, *σ*_G1_ = 0.27, *μ*_SG2M_ = 2.18, and *σ*_SG2M_ = 0.19 with mean values of 7.4 h and 9.0 h, respectively. Censored data (end of phase was not known or observable) and outliers are highlighted in red. (C-E) Correlations of phases in control cells. Similar to [20], the length of the cell cycle was plotted for individual cells against the respective length of the phases (C,D) and both phase lengths against each other (E). Pearson correlation coefficients (95% confidence interval) are as follows. (C): *ρ* = 0.83(0.82;0.84), (D): *ρ* = 0.8(0.76;0.83), (E) *ρ* = 0.33(0.24;0.42). (F,G) Tracking of cellular events in response to TRAIL. (F) Letters represent the following events in the exemplary video segments. a: G_1_ cell (geminin negative), b: S/G_2_/M cell (geminin positive), c: apoptosis, d: cell division. (G) Time of death (*t*_death_) box plots of cells treated with TRAIL in G_1_ (left panel) or S/G_2_/M (right panel). Boxes cover 25th to 75th percentiles. Median *t*_death_ for cells exposed in G_1_ was 2.9 h, and for cells in S/G_2_/M 3.5 h, indicated by red bars. Red crosses indicate outliers.

To allow for an analysis of potential links between cell cycle phases and cell death timing (Fig 1), we first characterized cell cycle progression in NCI-H460 cells expressing mAG-hGeminin(1/110) as a fluorescent reporter of S/G_2_/M phases [24]. Durations for G_1_ (geminin negative) and S/G_2_/M (geminin positive) phases were recorded for approximately 400 cells and described by lognormal distributions (Fig 2A and 2B). Lognormal distributions outperformed gamma, weibull and normal distributions in describing these data, judged from comparison of Bayesian information criteria (BIC) [26, 27] (S1 Table). This criterion was chosen because it takes model fit and complexity into account. Previous studies showed that S/G_2_/M phases were relatively constant and mainly variability in G_1_ caused different cell cycle times [28]. Our data provide a different picture: magnitudes of mean and variance were comparable for both phases (Fig 2A and 2B) and we observed a strong linear correlation of both phases with cell cycle durations (Fig 2C and 2D). The Pearson correlation coefficient of 0.33 for the durations of G_1_ and S/G_2_/M (Fig 2E) indicated a low linear correlation of the two processes. These findings are in line with [29] and [20], except for the cell line specific phase proportions of the total cell cycle durations (45% and 39% for G_1_ in NCI-H460 and HCT-116 (see S4 Fig), respectively).

Next, we studied cell fate and the time required for individual cells to execute cell death when treated with the EC50 dose of a hexavalent TRAIL receptor agonist [25] (Fig 1A). Approximately 550 G_1_ cells and 500 S/G_2_/M cells were monitored for at least 19 h prior to TRAIL addition to ensure that the mitotic history and thus age of each individual cell was known. The cell fate following TRAIL addition was recorded for another 19.25 h for randomly selected cells in the fields of view. Exemplary images are shown in Fig 2F. The probability of a cell to die when treated in G_1_ or S/G_2_/M was 0.76 and 0.72, respectively. At 95% confidence level the difference exceeded the maximal error calculated from the null hypothesis, indicating that the latter cells had a slightly increased likelihood to escape cell death induction. However, this difference was rather small and its functional significance is debatable. More interestingly, the time required until cells executed apoptosis (*t*_death_) differed substantially between cells exposed to TRAIL in G_1_ or S/G_2_/M phase, with S/G_2_/M cells requiring significantly longer to die (Δ median *t*_death_: 0.6 h, P-value: 4.0e-8, Fig 2G).

### TRAIL prolongs the durations of G_1_ and S/G_2_/M cell cycle phases

Next, we studied if TRAIL exposure affects the duration of cell cycle phases, irrespective of whether cell death is induced or not (Fig 1B). Cell death events might distort measurements by which drug effects on the duration of phases are to be determined [30, 31]. To test if censoring due to cell death plays a role in accurately determining the durations of cell cycle phases in our scenario, we first described cell cycle progression by a mathematical model for a virtual cell population. As in [32], we expressed the cell cycle *C* on a scale from 0 to 1, so that the G_1_ phase is represented by 0–0.45 and S/G_2_/M phase by 0.45–1. Values >1 correspondingly represent cells after mitosis. To define the virtual cell population that served as reference, the initial positions of cells in the cycle were sampled from an idealized steady state distribution, adapted from [33]

$$\begin{array}{c} F\left(C_{0}\right)=2^{1-C_{0}}\text{ln}\left(2\right). \end{array}$$ (1)

The position *C*^*i*^ in the cell cycle progresses for each individual cell *i*, allowing for individual growth rates *g*^*i*^ in G_1_ and S/G_2_/M phases so that

$$\begin{array}{cc} & \frac{dC^{i}}{dt}=g^{i},\;\;\text{ln}(\frac{f}{g^{i}})∼\{\begin{array}{cc} N(\mu_{G1},\sigma_{G1}^{2}), & \text{if}\;C_{i}\in [0,0.45[\;\text{or}\;C_{i}\in [1,1.45[,\\ N(\mu_{SG2M},\sigma_{SG2M}^{2}), & \text{if}\;C_{i}\in [0.45,1[\;\text{or}\;C_{i}\in [1.45,2[, \end{array} \end{array}$$ (2)

$$\begin{array}{cc} & f=\{\begin{array}{cc} 0.45, & \text{if}\;C_{i}\in [0,0.45[\;\text{or}\;C_{i}\in [1,1.45[,\\ 0.55, & \text{if}\;C_{i}\in [0.45,1[\;\text{or}\;C_{i}\in [1.45,2[, \end{array} \end{array}$$ (3)

$$\begin{array}{cc} & C^{i}\left(0\right)=C_{0}^{i},\;\;i\in \left[1,\ldots ,n\right]. \end{array}$$ (4)

where $\frac{f}{g^{i}}$ represents the durations of the respective cell cycle phases. Next, we assigned a representative cell death distribution to this virtual cell population, based on our experimental data of death times. To this end, we described the times required to die after TRAIL exposure (*t*_death_, Fig 2G) by a lognormal distribution

$$\begin{array}{c} \text{ln}\left(t_{\text{death}}^{i}\right)∼N\left(\mu_{d},\sigma_{d}^{2}\right),\;\;i\in \left[1,\ldots ,n\right], \end{array}$$ (5)

taking into account that sibling cells dying after mitosis commit synchronous apoptosis shortly after mitosis [12, 17]. As such, cell death events after mitosis (f1 generation) were not inappropriately over-weighted. With help of Eqs 1–5, the cell cycle positions at the time of death were calculated with

$$\begin{array}{c} C^{i}\left(t_{\text{death}}^{i}\right)=C_{0}^{i}+g^{i}\;t_{\text{death}}^{i},\;\;t^{i}\left(0\right)=t_{\text{TRAIL}}^{i}=0,\;\;C^{i}\left(0\right)=C_{0}^{i},\;\;i\in \left[1,\ldots ,n\right]. \end{array}$$ (6)

**Fig 3:**
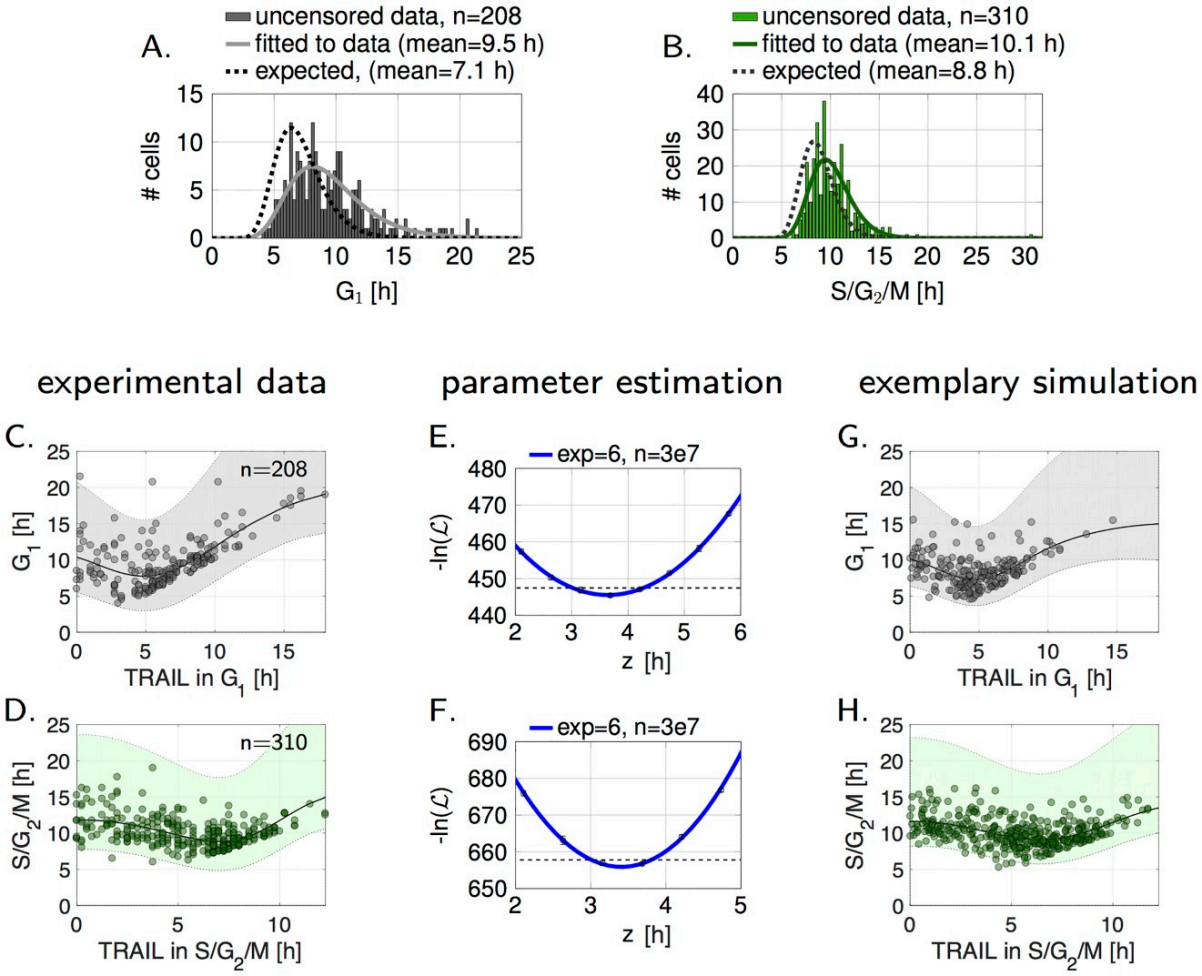
Lengths of G_1_ and S/G_2_/M phases in response to TRAIL. (A,B) Lengths of G_1_ and S/G_2_/M phases of NCI-H460 cells that survived the phase of TRAIL exposure. The expected distributions were approximated under consideration of the fraction of surviving and uncensored, apoptotic cells. (C,D) Experimentally determined correlations of phase length and time of TRAIL addition. A linear gaussian process was assumed to highlight the 95% confidence interval. One outlier (S/G_2_/M > 25 h) was omitted from the display (D). (E,F) Negative log-likelihood for varying prolongations of phases (*z*-values). 6 experiments with 3e7 samples were conducted. Mean and standard deviations are shown and a polynomial of 5th grade was fitted to the resulting mean. The lowest negative log-likelihood values were obtained for $\hat{z}=3.6\;\text{h}$ (3 h-4.3 h), and $\hat{z}=3.4\;\text{h}$ (3 h-3.8 h), respectively. Confidence bounds are indicated with dashed lines. (G,H) Exemplary model simulations with $\hat{z}$-values are shown. The 95% confidence intervals were calculated with a linear gaussian process and highlighted in gray and green.

We next compared the duration of G_1_ and S/G_2_/M phases between control cells and a cell population dying after TRAIL using this model. Cells that progressed through their cell cycle phase without dying first (*C*(*t*_death_) > *f*) do so faster than cells in the control population (approximately 0.5 h and 0.3 h for G_1_ and S/G_2_/M phases, respectively) (S1 Fig). This can be explained by removing cells that die before phase end in these types of analyses. Profiles of faster cell death distributions augment these effects further (S2 Fig). To accurately compare experimentally measured phase durations following TRAIL treatment to durations in untreated cells, we considered these time shifts. As seen in Fig 3A and 3B, TRAIL treatment prolonged both, G_1_ and S/G_2_/M phases, by 2.4 h and 1.3 h, respectively. For validation, we showed that the experimentally determined G_1_ durations in cells prior to TRAIL exposure was indistinguishable from the control distribution (S3 Fig). Next, we investigated if the prolongation of cell cycle phases is linked to the duration from phase start to TRAIL exposure (*d*^*i*^) within G_1_ or S/G_2_/M phases (Fig 3C and 3D). Comparing the total phase duration against *d*^*i*^ revealed a negative slope during the first 4-5 h, indicating that the earlier a cell was stimulated in its current phase, the more pronounced was phase prolongation. As cells receiving TRAIL at later time points already exhibit longer phase durations, increasing values at later times were expected. Consequently, the most simplistic assumption is that TRAIL decelerates cell cycle progression in apoptosis competent cell populations. This can be expressed by introducing a prolongation term *z* for the respective cell cycle phases, resulting in a corrected growth rate

$$\begin{array}{c} g^{i,*}=\frac{f}{\frac{f}{g^{i}}+z},\;\;\;i\in \left[1,\ldots ,n\right],\;\;\;C_{0}^{i}\in [0,1[. \end{array}$$ (7)

To determine *z*, we conducted a parameter estimation (for detailed description see method section) by comparing phase durations of experimental data to large sample populations that were constructed according to Eqs 1–7 (Fig 3E and 3F). Best values $\hat{z}$ describing the experimental data were 3.6 h (3-4.3 h, 95% confidence interval) and 3.4 h (3-3.8 h, 95% confidence interval), respectively. Simulating G_1_ and S/G_2_/M durations based on these values provided distributions very similar to the experimental results (Fig 3G and 3H, compare to Fig 3C and 3D). This confirms that the mathematical model describes the influence of TRAIL on cell cycle progression sufficiently well. For the cell line HCT-116, we observed a similar behaviour, with slightly lower values $\hat{z}$ (2.5 h (2.1-3 h), and 1.95 h (1.4-2.5 h) for G_1_ and S/G_2_/M, respectively) (S5 Fig).

### The time of TRAIL exposure in S/G_2_/M phase appears linked to the likelihood of apoptotic cell death

**Fig 4:**
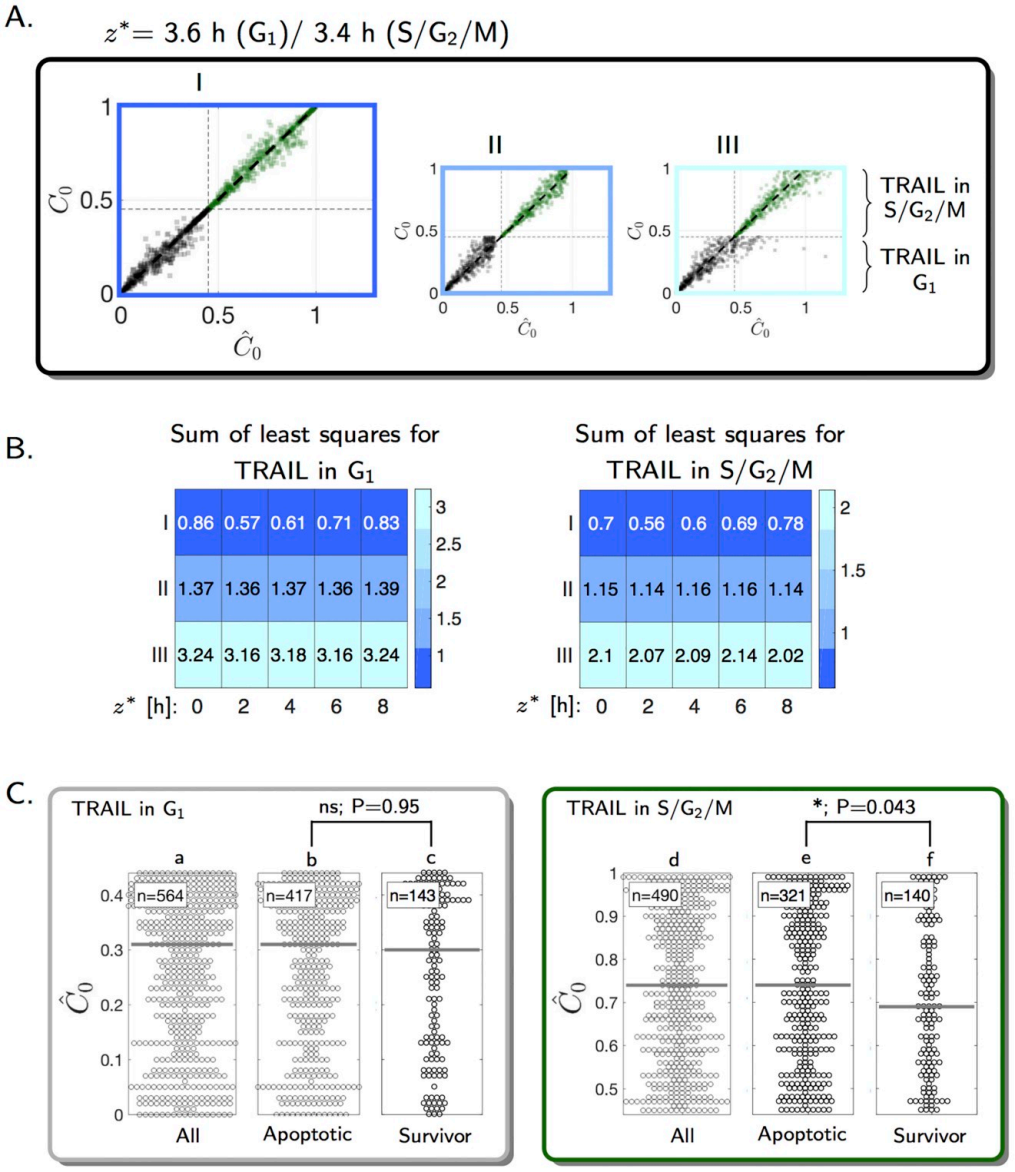
Estimation of the cell cycle position at TRAIL exposure and its impact on cell fate. (A) *C*_0_ indicates the real initial cell cycle position whereas $\hat{C_{0}}$ represents estimated values. For calculating $\hat{C_{0}}$, the approximation of the initial cell cycle position, a data set of a virtual cell population was normalized with a sample of (III) the original distribution of G_1_ or S/G_2_/M phases or (II) the respective truncated distributions. In (I), either the truncated distribution was used for normalization or, for cells that completed the phase, $\hat{C_{0}}$ was calculated as a function of $\hat{z}$ and the length of the phase. (B) Indicated values of z* were assumed for constructing the virtual cell population. Sum of least squares $\sum_{i=1}^{n}{\left({\hat{C}}_{0}-C_{0}\right)}^{2}$ are indicated for the different scenarios. (C) Impact of initial cell cycle positions on cell fate in experimental data. Gray bars represent median values. Apoptotic: cell death before division or both daughter cells died. Survivor: both daughter cells survived. Mixed: 2 in G_1_ and 29 in S/G_2_/M. P-values according to Wilcoxon rank sum test [35] are as follows. b/c: 0.95 (not significant, ns), e/f: 0.043 (significant, *).

Next, we studied if the position of a cell within the G_1_ or S/G_2_/M phases at the time of exposure to TRAIL affects the probability and timing of cell death (Fig 1D I). To do so, we developed an approach to estimate the position of individual cells within the cell cycle reliably (Fig 1C). Therefore, we took into account that the durations of the cell cycle phases are heterogeneous across the population, that apoptosis events impede determining the end point of a phase in a fraction of the cell population, and that TRAIL exposure itself alters the progression of the cell cycle (see Fig 3). The concept of our approach was initially tested on a virtual cell population consisting of *i* cells with proliferation rates according to Eq 7 and a correction term z*. The initial cell cycle positions $C_{0}^{i}$ were sampled from a standard uniform distribution and death time ($t_{\text{death}}^{i}$) was sampled according to Eq 5. We extracted the following information from the virtual cell population: i) the duration from start of the cell cycle phase to TRAIL addition (*d*^*i*^), ii) the cell cycle position at time of cell death *C*(*t*_death_), and iii) the duration of the cell cycle phase (*p*^*i*^) for cells that failed to die within this phase. We then tested if the initial cell cycle positions $C_{0}^{i}$ of cells in this virtual population can be estimated by

$$\begin{array}{cc} & {\hat{C}}_{0}^{i}=\{\begin{array}{cc} \frac{1}{m}\sum_{j=1}^{m}\frac{f\;d^{i}}{p_{u}^{j}},\;\;p_{u}^{j}∼\text{pdf}\left(p_{u}|p_{u}>d^{i}\right), & \text{if}\;C_{\text{death}}^{i}\leq f,\\ h(p^{i},d^{i},\hat{z}), & \text{if}\;C_{\text{death}}^{i}>f, \end{array} \end{array}$$ (8)

$$\begin{array}{cc} & j\in [1,\ldots ,m],\;\;i\in [1,\ldots ,n], \end{array}$$ (9)

where *f* represents the respective value of G_1_ and S/G_2_/M phases (compare to Eqs 2 and 3 and associated Fig 2A and 2B) and pdf(*p*_*u*_) represents the distribution of phase lengths in unstimulated cells (see Eq 2). For cells that died after the end of the cell cycle phase in which they were treated, a sample of a truncated distribution was used for normalization of the cell cycle position. This allowed taking into account that such cells already spent a defined time within the respective cell cycle phase, prior to TRAIL treatment and induction of cell death. In cells that continued to proliferate, the cell cycle position ${\hat{C}}_{0}^{i}$ was calculated as a function of $\hat{z}$, the estimated TRAIL-induced cell cycle prolongation, the duration of the respective phase *p*^*i*^, and the time of TRAIL addition *d*^*i*^ (Eq 8). Our estimations of the cell cycle positions ${\hat{C}}_{0}^{i}$ agreed very well with the real position $C_{0}^{i}$ of the virtual population (Fig 4A(I)). In contrast, estimations based solely on normalization with samples of the truncated population (Fig 4A(II)) or the unstimulated population (Fig 4A(III)) were more error prone. Reliable estimations of cell cycle positions were also possible for alternative correction terms z* for the virtual cell population (Fig 4B). Our approach is therefore suitable for arranging cells according to their cell cycle position. Consequently, we next applied this to experimental data to asses if initial cell cycle positions influence cell fate. When NCI-H460 cells were stimulated with TRAIL in G_1_, cell fate was independent of the initial cell cycle position (Fig 4C). With respect to S/G_2_/M, cells exposed to TRAIL earlier were more likely to survive (P-value: 0.043, Fig 4C).

### Model analysis describes apoptosis deceleration in S phase

**Fig 5:**
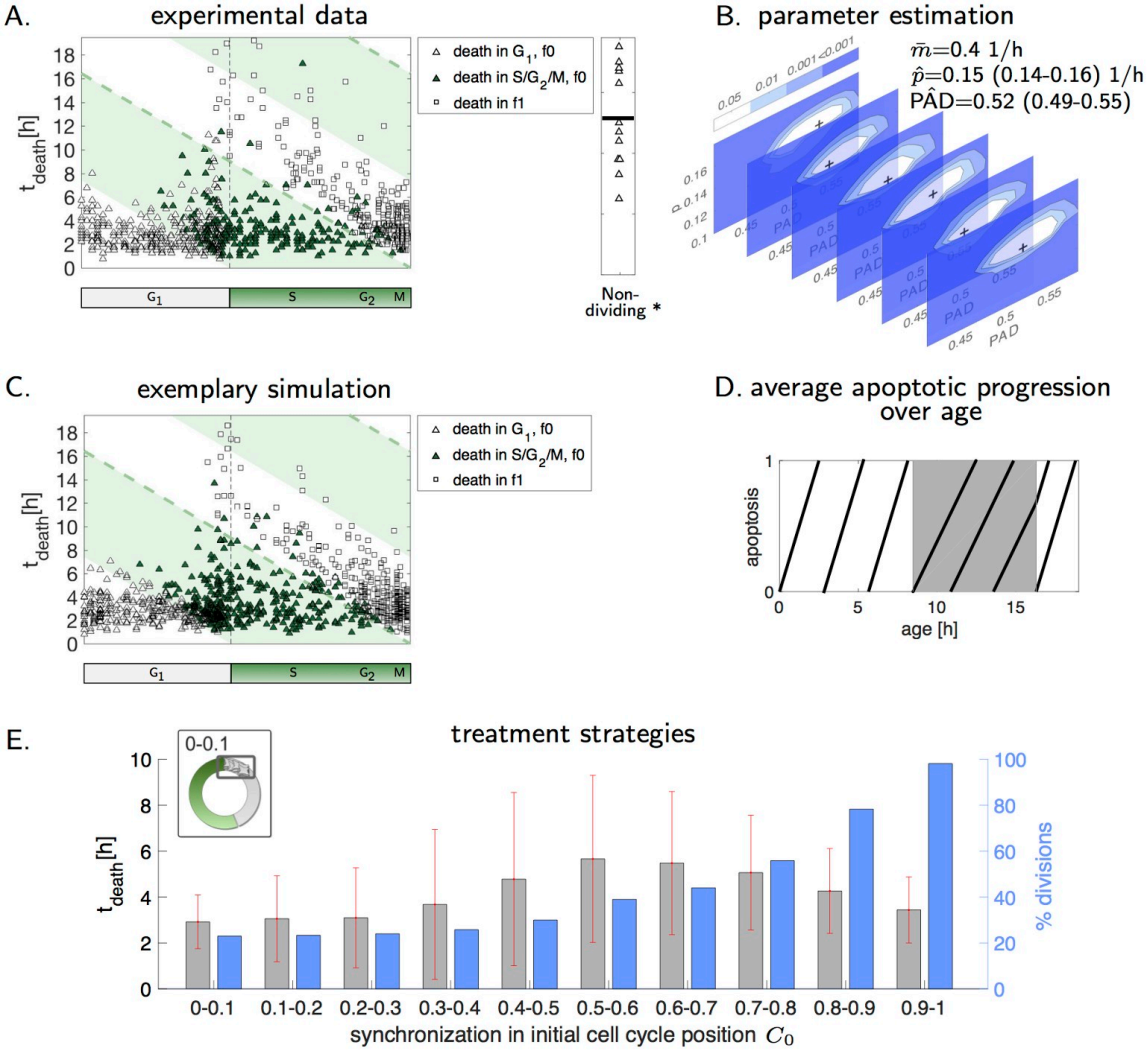
Timing of death depends on cell cycle positions. (A) Death times in experimental data (n = 921) were plotted against estimated initial cell cycle positions. Symbols indicate cell cycle phases at time of cell death. Green shading indicates S/G_2_/M phase and dashed line represents division of an averaged, untreated NCI-H460/geminin cell. (B) Parameter estimation was conducted six times with independent samples of initial model conditions. The sample size for each simulation experiment was approximately 8e4. Significance levels are shown by color coding. Indicated values of $\hat{p}$ and $\hat{\text{PAD}}$ are mean and extreme values of 6 experiments. (C) An exemplary model-based simulation with best parameter values $\hat{p}$ and $\hat{\text{PAD}}$ is shown. (D) Average apoptotic progression in dependence on the ages of cells, representing the time from birth to TRAIL addition, is illustrated. The area of decelerated apoptosis progression is highlighted in gray. (E) Different synchronization strategies (*C*_0_ ∈ (0, 0.1), *C*_0_ ∈ (0.1, 0.2),…) impact death timing and number of divisions.

Next, we analyzed if the cell cycle position at the time of TRAIL addition influences the time between TRAIL exposure and cell death (Fig 1D II). Interestingly, when plotting the estimated cell cycle position at TRAIL exposure against the time to death (*t*_death_) for each individual cell (Fig 5A), the population divides. Cells stimulated in late G_1_ or early S phase, either died in f0 with death times comparable to stimulation in early G_1_, or alternatively with a significantly prolonged death time after the subsequent mitosis. Prolonged death times disappeared in cells exposed to TRAIL close to mitosis (Fig 5A). To identify the cell cycle position at which apoptosis suppression manifests, we developed a phenomenological model. The model consists of Eqs 2–4 and an additional, coupled ordinary differential equation describing apoptosis progression

$$\begin{array}{cc} & \frac{dA^{i}}{dt}=m^{i}-p,\;\;\;p=\{\begin{array}{cc} 0, & \text{if}\;C^{i}\in \left[0,\text{PAD}\right],\\ p, & \text{else,} \end{array} \end{array}$$ (10)

$$\begin{array}{cc} & t^{i}\left(0\right)=t_{\text{TRAIL}}^{i}=0,\;\;C^{i}\left(0\right)=C_{0}^{i}, \end{array}$$ (11)

$$\begin{array}{cc} & A\left(0\right)=0,\;\;\text{min}\left(A\left(t\right)\right)=0,\;\;A\left(t_{\text{death}}^{i,M}\right)=1, \end{array}$$ (12)

$$\begin{array}{cc} & y\left(p,\text{PAD}\right)=-\text{ln}L(t_{\text{death}}^{M}\left(p,\text{PAD}\right)|t_{\text{death}}^{D}), \end{array}$$ (13)

$$\begin{array}{cc} & \hat{p},\hat{\text{PAD}}=\underset{p,\text{PAD}}{\text{arg}\;\text{min}}\left(y\left(p,\text{PAD}\right)\right). \end{array}$$ (14)

Apoptosis progresses over time with a constant slope *m* and *p* represents inhibition of apoptosis progression. PAD (Point of Apoptosis Deceleration) refers to the unknown point in the cell cycle after which apoptosis progression will be delayed. M and D emphasize model and experimental death times, respectively. Since molecularly the delay in apoptosis execution might be related to cell cycle-regulated changes in protein amounts, the delay or inhibition of signal propagation is removed after cell division. First, we approximated *t*_death_ of early G_1_ cells with a lognormal distribution (*μ*_*td*_ = 1, *σ*_*td*_ = 0.39), resulting in an average death time of 2.9 h in these cells. *m*^*i*^ (Eq 10) is the inverse of a sample of this distribution ($\bar{m}=0.4$ 1/h). Next, the negative log-likelihood of death times in dependence on the initial cell cycle position *C*_0_ (Eq 13) for different values of *p* and PAD was used to calculate corresponding significance levels and approximate best parameters $\hat{p}$ and $\hat{\text{PAD}}$ (Eq 14) (for detailed description see method section). We conducted these computations for six independent sampling experiments, all of which provided similar best points and confidence regions of $\hat{p}$ and $\hat{\text{PAD}}$ (Fig 5B). Based on 95% confident intervals of PAD and *p*, the time point of apoptosis deceleration was reached at around 49% to 55% of the cell cycle. We therefore conclude that apoptosis progression was delayed approximately in mid S phase. Importantly, apoptosis progression was not fully blocked, since slope $\bar{m}>\hat{p}\;\forall \;C^{i}$ (Fig 5B). An example simulation based on the estimated parameters indeed reproduced the experimental data very well (Fig 5C). In Fig 5D, the idealized courses of apoptosis progression in relation to cell age are visualized, where young cells die faster than older cells upon TRAIL exposure. When comparing these results to different “null” models in which cell cycle-independent distributions were fitted to the death kinetics, substantially larger values for the Bayesian information criterion were obtained (S2 Table). These data further support cell cycle dependence of extrinsic apoptosis progression. Although cell death was slower in the case of the cell line HCT-116, parameter estimation suggests a similar point of apoptosis deceleration at the end of G_1_ or beginning of S-phase (S6 Fig).

Since cells that proceed through mitosis as a consequence of apoptosis suppression ultimately might escape cell death, we conceptually studied if and which cell cycle synchronization strategies would maximize rapid apoptosis execution in cell populations. In a virtual cell population constructed from the presented experimental results, we analyzed how synchronization within G_1_ or S/G_2_/M affects *t*_death_ and the number of cells that go through mitosis (Fig 5E). Cells died most rapidly when treated in the first 30% of the cell cycle. In contrast, TRAIL exposure between 50% to 70% of the cell cycle resulted in the longest and most variable death times (Fig 5E). Correspondingly, pre-treatment with the FDA-approved CDK4/6 inhibitor Abemaciclib synchronized NCI-H460/geminin cells in G_1_ phase and significantly reduced the death time in response to TRAIL (S7 Fig). Overall, these data therefore demonstrate that apoptosis progression is suppressed in favor of mitosis by mid S phase and that optimal treatment responsiveness can be expected for cells exposed to TRAIL early after division.

## Discussion

In this study we combined microscopic fluorescence-based monitoring of cell cycle progression and cell death execution with sampling-based mathematical modeling to study if extrinsic apoptosis signaling in NCI-H460/geminin cells is influenced by cell cycle progression. Interestingly, we found that cell cycle progression, in particular transitioning through S phase, significantly prolongs the time required for cells to die in response to TRAIL. In a companion study, we found that these delays suffice to let sublethally damaged cells escape from apoptosis [36]. In reverse, TRAIL addition prolonged G_1_ and S/G_2_/M phases, a previously unreported phenomenon. Our study is the first to systematically and quantitatively analyze the interdependence of cell cycle progression and TRAIL-induced apoptosis without exteriorly interfering with either of these processes during the course of the analyses. This could be achieved by integrating non-invasive time-lapse imaging with mathematical modeling and parameter estimation. As experimental data sources, our study strongly relied on determining accurate kinetics for cell cycle progression and the times required for TRAIL to induce apoptosis execution. Prolonged time-lapse imaging, coupled with information on the mitotic history of the analyzed cells and fluorescence-based cell cycle phase monitoring, allowed us to confidently estimate cell cycle positions at minutes resolution. In addition, population heterogeneity in cell cycle synchrony could be taken into account within the modeling and parameter estimation framework. Our approach therefore circumvented the limitations imposed by conventional cell synchronization in experimental studies, such as arresting and releasing cells in distinct cell cycle phases, which would have prevented analyzing signaling interplay during regular cell cycle progression [37].

Indeed, conditions where cells are arrested in or released from distinct cell cycle phases can easily lead to the misinterpretation of results on TRAIL sensitivity, since cell cycle arrest in itself sensitizes to apoptosis [38]. Consequences on apoptosis susceptibility in such settings might therefore be independent of cell cycle progression. For example, tumor suppressor p53 plays a crucial role in cell cycle arrest but also induces the expression of various apoptosis proteins [6, 39], which in turn can synergize with TRAIL-induced caspase 8-dependent cell death. Despite the interesting insight such studies provide for TRAIL response potentiation, a comprehensive understanding of the interrelation between cell cycle progression and susceptibility to TRAIL-induced apoptosis can only be obtained by the approach chosen here, using unsynchronized cell populations. However, also studies in which cells were not artificially synchronized reported inconsistent findings for the relationship between extrinsic apoptosis susceptibility and cell cycle progression [12, 38, 40–42]. One problem in “observation only” studies arises from neglecting the possibility of bidirectional relationships between cell cycle progression on TRAIL responsiveness and TRAIL exposure on cell cycle progression, both of which we identified in our study. This aspect is non-trivial, since cells dying during the course of treatment pose a significant problem for the cell cycle analysis. We solved this problem by integrating mathematical modeling and parameter estimation into the workflows. To our knowledge, phenomenological mathematical models that define connections between the cell cycle and extrinsic apoptosis progression have not been reported yet. However, model-based studies that combine proliferation and cell death exist [43, 44]. In larger scale mechanistic models, extrinsically stimulated cellular signaling processes were implemented together with abstracted cell cycle signaling [21, 22], however, their complexity would impose parameter estimation to become computationally highly expensive and problems in parameter identifiability would arise. Focusing solely on a phenomenological description of the interconnection between cell cycle and cell death instead allowed us to appropriately estimate parameters and obtain novel biological insight. This might serve as a basis on which more complex mechanistic models could be implemented to dissect the molecular mechanisms that underlie the observed effects. The approach chosen here can likewise serve as a template to study other cell biological processes and their potentially bidirectional relationship to cell cycle progression.

## Materials and methods

### Time-lapse microscopy and cell tracking

Establishment of the NCI-H460/geminin cell line was described in [24]. To observe cell cycle history and cell death kinetics, cells were grown on 35 mm glass-bottom dishes (CellView Cell Culture Dish, Greiner Bio One) in phenol red free RPMI 1640 containing 10% FBS. Time-lapse images were acquired every 15 minutes on a Zeiss Cell Observer microscope equipped with a Plan-Apochromat 20x/0.8 objective at 37°C, 5% CO_2_. Fluorescence of mAG-hGeminin(1/110) was acquired with a 470 nm LED module combined with a 62 HE filter set (Carl Zeiss). After 19 h, Fc-scTRAIL (0.06 nM, [25]) was added and cells were imaged for another 19.25 h. Images were processed using the ZEN2.1 blue software (Carl Zeiss). Randomly chosen cells were tracked manually and the time of birth, onset of geminin expression (representing entry into S phase), cell division and the time from TRAIL addition to death (*t*_death_) was recorded. A cell was assigned as geminin positive as soon as geminin expression was above the background. Geminin positive cells were assigned to the combined S/G_2_/M phases. A cell was assigned to be apoptotic as soon as cell blebbing was visible.

With respect to the experiments shown in S7 Fig, NCI-H460/geminin cells were pre-treated with DMSO or Abemaciclib (2 *μ*M, Selleckchem) for 3 h. Then, medium containing Fc-scTRAIL was added and cells were imaged for another 10 h. Randomly chosen cells were tracked and *t*_death_ of cells was recorded.

### Modeling and statistical analysis

For calculations, MATLAB (MATLAB R2017a, The MathWorks, Natick, 2017) and the Statistics and Machine Learning Toolbox were used. For computing the Bayesian information criteria (BIC) [26, 27], the length of phases that were not completely measurable was included as censored information. In order to approximate expected distributions for apoptotic cells (S1 Fig), independent samples of *C*_0_ (Eq 1) and experimentally determined *t*_death_ (Eq 5) distributions were drawn. $C^{i}\left(t_{\text{death}}^{i}\right)$ was calculated (Eq 6) and cells with $C^{i}\left(t_{\text{death}}^{i}\right)\leq f$ were discarded. A lognormal distribution was fitted to the remaining phase lengths.

For approximating expected distributions (Fig 3A and 3B), the experimentally determined ratio of surviving and apoptotic cells was taken into account. The probability that two distributions of interest are samples from underlying distributions with the same median was estimated with help of the Wilcoxon rank sum test [35]. Except for results shown in S6 Fig, ordinary differential equations were solved analytically. For S6 Fig, the Matlab toolbox IQM Tools [46] was used. Computational methods and unprocessed data are available at Github: https://github.com/DirkeImig/CellCycleApoptosis.

#### Parameter estimation

For parameter estimation of correlations between length of phases and time of TRAIL addition *d*^*i*^ (Fig 3E and 3F), the following algorithm was used: First, representative samples of **C**_**0**_, **t**_death_ and **g** were drawn to construct a virtual cell population. Surviving cells ($t_{\text{death}}^{i}=\text{inf}$) were added according to the fraction of surviving cells that was observed experimentally. Cells that died before reaching the end of the respective phase ($C^{i}\left(t_{\text{death}}^{i}\right)<f$, Eq 6) were discarded. Next, *d*^*i*^ was calculated (Eqs 2–4) for the remaining cells. Then, the overall lengths of the phases were computed (Eqs 2–4 with *g*^*i*^ = *g*^*i*,*^, Eq 7). A lognormal distribution was fitted to the vector of model phase lengths that correspond to a specific time of TRAIL addition and the respective likelihood of describing the experimental data was evaluated. Lastly, the sum of all negative log-likelihood values was calculated. Negative log-likelihood values for different *z*−values of 6 separate simulation experiments with 5e7 sampled cells were averaged and shown in Fig 3E and 3F. The minimum value of the negative log-likelihood revealed the best parameter estimate $\hat{z}$.

For parameter estimation of death kinetics (Fig 5B), first, representative samples of **C**_0_, **g** and **m** were drawn and $t_{\text{death}}^{M}$ was calculated for given values of *p* and PAD (Eqs 10–14). M and D represent model and experimental data, respectively. We approximated the bivariate model density of **C**_0_ and $t_{\text{death}}^{M}$ with help of a kernel density estimator. For each data tuple {$C_{0}^{j,D}$,${t^{j,D}}_{\text{death}}$}, the likelihood of being described by the model was evaluated and the overall negative log-likelihood was calculated. The process was repeated for different values of PAD, and *p*, originated from a predefined grid in the parameter space. To avoid rounding errors, the lowest value within the density was set to 1e-9. Confidence intervals were calculated according to *χ*^2^ statistics [34].

## Supporting information

S1 Table Information criteria for different distributions. The negative log-likelihood and respective BIC values are shown for the indicated model distributions. The number of parameters equals 2 for lognormal, weibull and normal distributions and 4 in case of the gamma distribution. A lower BIC indicates a better model fit, with a difference higher than 2 being positive in evidence [45].
(TIF)

S2 Table Δ BIC for different null models. Differences of BIC values between null models with indicated distributions and best BIC for the model in Eqs 10–14 is shown. A Δ in BIC > 2 means positive in evidence [45]. Here, all differences are significant.
(TIF)

S1 Fig Expected distributions of G_1_ and S/G_2_/M in apoptotic cells. A sample of the fitted lognormal distribution of untreated cells is shown in black (G_1_, A) and green (S/G_2_/M, B). In white, lengths in the modeled virtual population are shown, given *C*(*t*_death_) > *f* (Eq 3).
(TIF)

S2 Fig Influence of different *t*_death_ distributions on expected distributions of uncensored cells. Different values for *μ* and *σ* regarding the lognormal *t*_death_ distributions are evaluated. The mean differences between original distributions and calculated values given that *C*(*t*_death_) > *f* (Eq 3) are shown. The lower *μ*, the greater the difference between original and approximated distribution. Reason for this is that with a faster death impulse, the likelihood of reaching the end of a phase is lower for cells with relatively long phase lenghts. The underlying phase lengths distribution corresponds to G_1_ phase lengths in control cells. This is similarly valid for S/G_2_/M phases.
(TIF)

S3 Fig Length of G_1_, f0 phase for cells treated in S/G_2_/M. In order to exclude a possible influence of experimental conditions on phase length variability, the G_1_, f0 phase lengths of cells that were treated with TRAIL in S/G_2_/M (n = 446) was compared to the control case. With a P-value of 0.54 [35], the null hypothesis of the two distributions originating from a distribution with the same median could not be rejected.
(TIF)

S4 Fig Length of G_1_ and S/G_2_/M in control HCT-116 cells. (A,B) Parameters of underlying lognormal distributions are *μ*_G1_ = 1.87, *σ*_G1_ = 0.34 and *μ*_SG2M_ = 2.34, *σ*_SG2M_ = 0.22 with mean values of 6.9 h and 10.7 h. Censored data (end of phase is not known) and outliers are highlighted in red.
(TIF)

S5 FigLengths of G1 and S/G2/M phases in HCT-116 in response to TRAIL.(A,B) Experimentally determined correlations of phase length and time of TRAIL addition. A linear gaussian process was assumed to highlight the 95% confidence interval. (C,D) Negative log-likelihood for varying prolongations of phases (z-values). 3 experiments with 3e6 samples were conducted. Mean and standard deviations are shown and a polynomial of 5th grade was fitted to resulting mean values. The lowest negative log-likelihood values were obtained for $\hat{z}=2.5\;\text{h}$ (2.1 h–3 h), and $\hat{z}=1.95\;\text{h}$ (1.4 h–2.5 h), respectively. Confidence bounds are indicated with dashed lines. (E,F) Exemplary model simulations with $\hat{z}$-values are presented. The 95% confidence intervals were calculated with a linear gaussian process, highlighted in gray and green.(TIF)

S6 FigTiming of death depends on cell cycle positions in HCT-116.(A) Death times of experimental data (n = 538) were plotted against estimated initial cell cycle positions. Symbols indicate cell cycle phases at time of cell death. Experiment time was 24.5 h. Green shading highlights S/G_2_/M phase and the dashed line represents division of an averaged, untreated HCT-116/geminin cell. (B) Parameter estimation with six independent samples of initial conditions. Each sample size was approximately 1e4. *m* was estimated from cells dying in early G_1_. For simulation, the Matlab toolbox IQM Tools [46] was used and PAD was extended for the second generation so that *p* = 0 if *C*^*i*^ ∈ [0, *PAD*] or [1, *PAD* + 1] (see Eq 10). Significance levels are shown by color coding. Values of $\hat{p}$ and $\hat{\text{PAD}}$ represent mean and extreme values of six simulation experiments. (C) An exemplary simulation with best parameter values $\hat{p}$ and $\hat{\text{PAD}}$ is shown. (D) Average apoptotic progression in dependence on the ages of cells, representing the time from birth to TRAIL addition, is illustrated. The area of decelerated apoptosis progression is highlighted in gray.(TIF)

S7 Fig Cell death after inhibition of CDK4/6 in NCI-H460/geminin cells. A. Representative time-lapse images of NCI-H460/geminin cells treated with Fc-scTRAIL (0.06 nM) or Abemaciclib (2 *μ*M). Scale bar represents 50 *μ*m. Dying cells are highlighted by white arrowheads. B. Times required to die (*t*_death_) of NCI-H460/geminin cells following treatment with Fc-scTRAIL alone or after pre-treatment with Abemaciclib (2 *μ*M, 3 h). Shown are medians with interquartile ranges, plus min to max range from n = 50 cells observed in three independent experiments (**** p < 0.0001, unpaired t-test). Na = not applicable.
(TIF)
