# Supplementary figures and images for: Sample-based modeling reveals bidirectional interplay between cell cycle progression and extrinsic apoptosis
Source: PLoS Comput Biol. 2020 Jun 4;16(6):e1007812. doi: 10.1371/journal.pcbi.1007812 (PMC7271993; doi:10.1371/journal.pcbi.1007812)

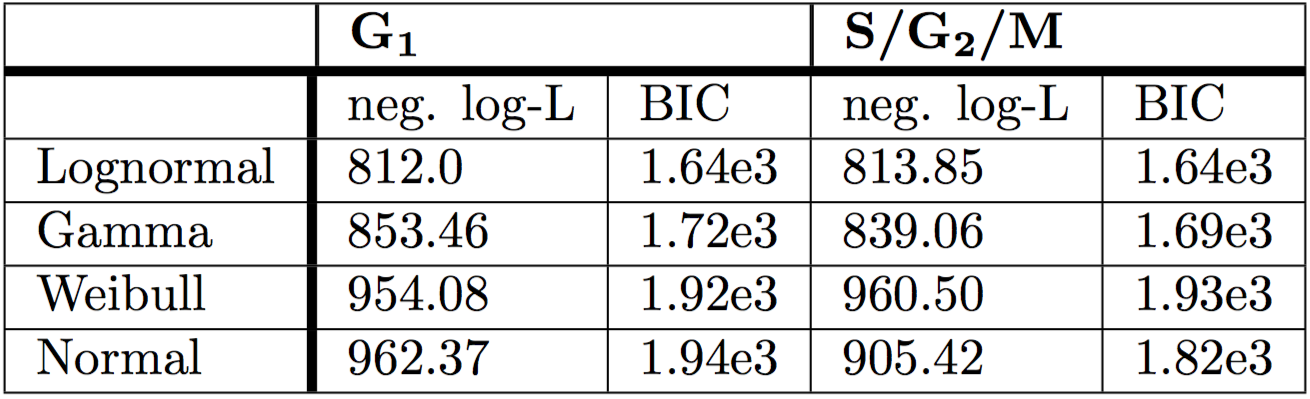

Supplement: S1 Table — The negative log-likelihood and respective BIC values are shown for the indicated model distributions. The number of parameters equals 2 for lognormal, weibull and normal distributions and 4 in case of the gamma distribution. A lower BIC indicates a better model fit, with a difference higher than 2 being positive in evidence [45]. (TIF) [file pcbi.1007812.s001.tif]

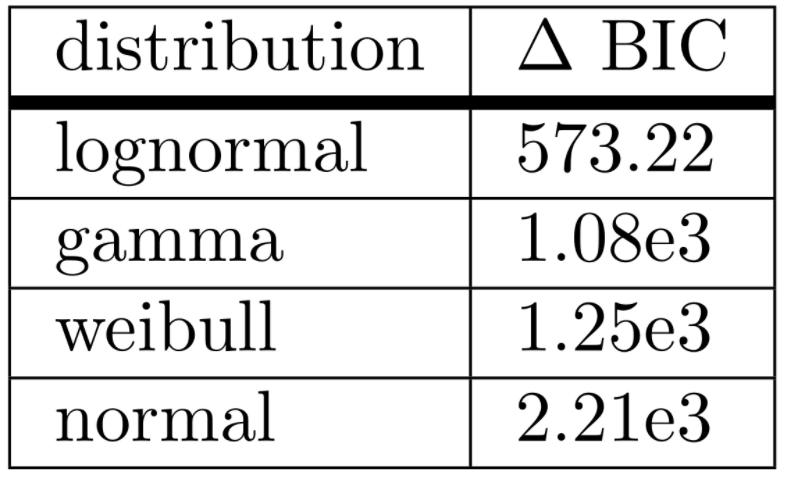

Supplement: S2 Table — Differences of BIC values between null models with indicated distributions and best BIC for the model in Eqs 10–14 is shown. A Δ in BIC > 2 means positive in evidence [45]. Here, all differences are significant. (TIF) [file pcbi.1007812.s002.tif]

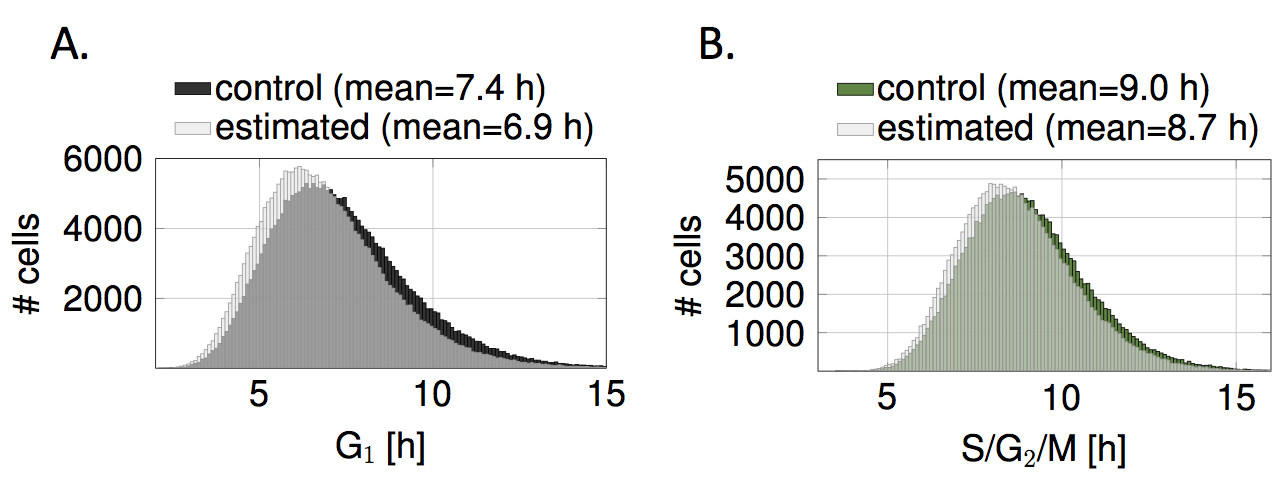

Supplement: S1 Fig — A sample of the fitted lognormal distribution of untreated cells is shown in black (G1, A) and green (S/G2/M, B). In white, lengths in the modeled virtual population are shown, given C(tdeath) > f (Eq 3). (TIF) [file pcbi.1007812.s003.tif]

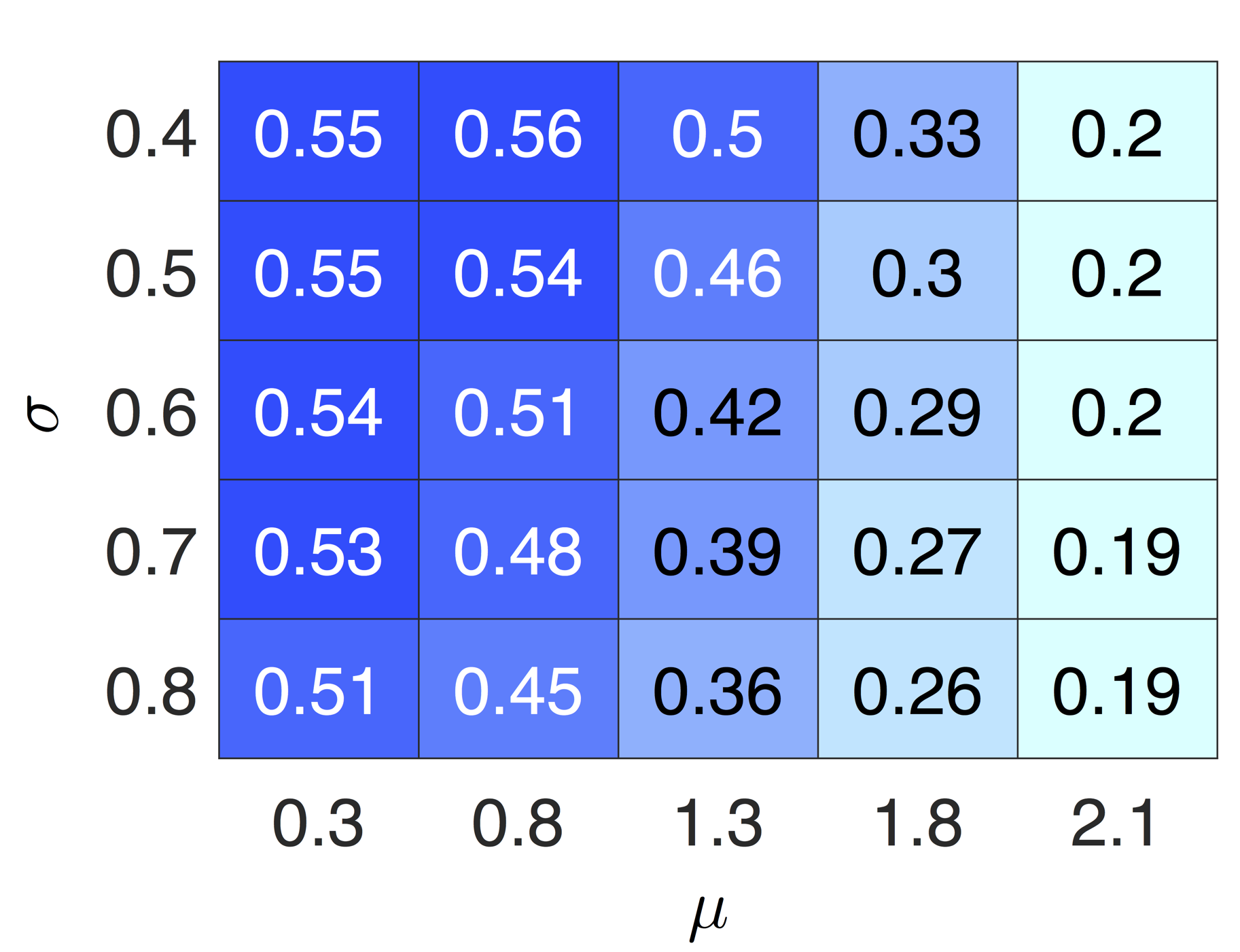

Supplement: S2 Fig — Different values for μ and σ regarding the lognormal tdeath distributions are evaluated. The mean differences between original distributions and calculated values given that C(tdeath) > f (Eq 3) are shown. The lower μ, the greater the difference between original and approximated distribution. Reason for this is that with a faster death impulse, the likelihood of reaching the end of a phase is lower for cells with relatively long phase lenghts. The underlying phase lengths distribution corresponds to G1 phase lengths in control cells. This is similarly valid for S/G2/M phases. (TIF) [file pcbi.1007812.s004.tif]

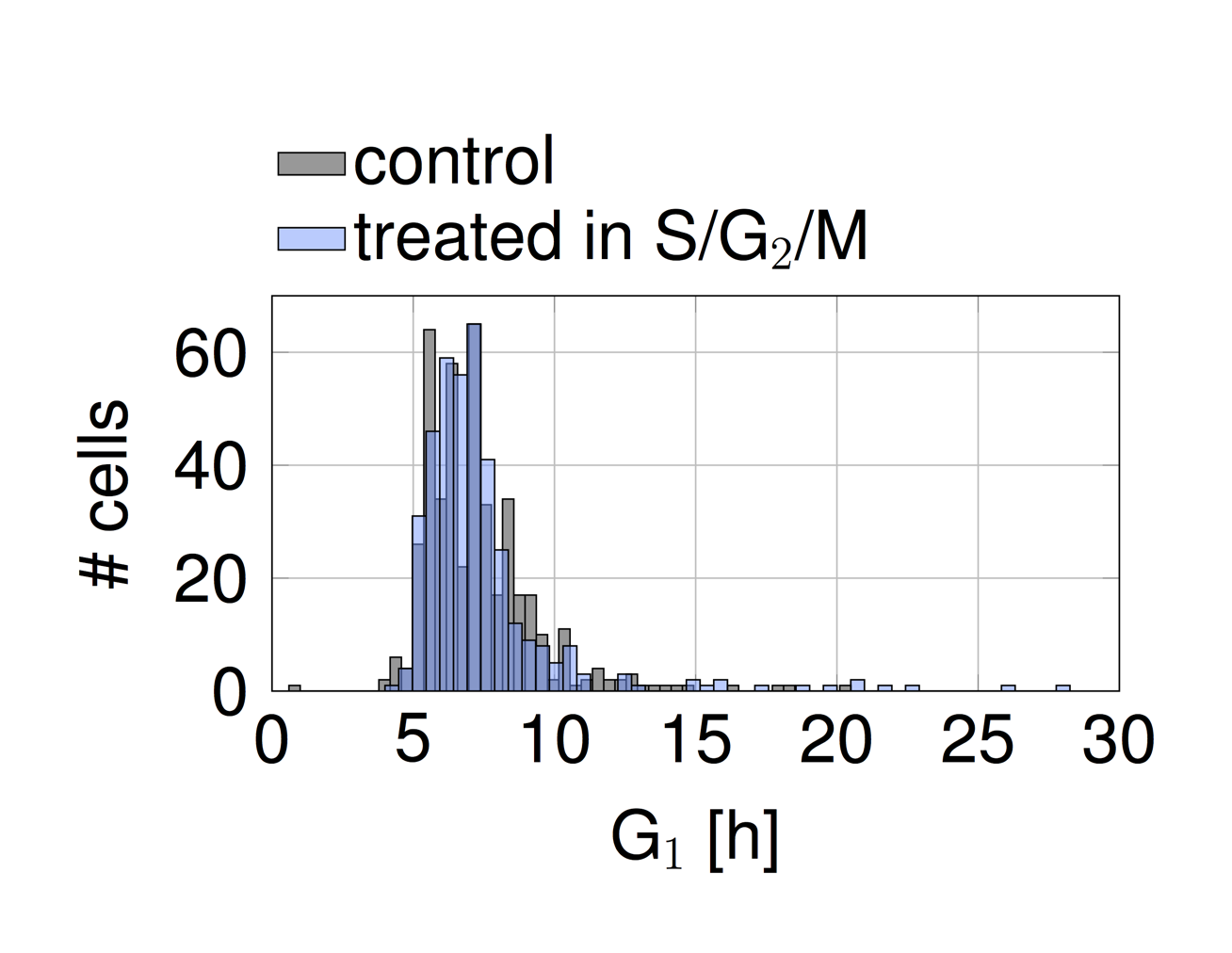

Supplement: S3 Fig — In order to exclude a possible influence of experimental conditions on phase length variability, the G1, f0 phase lengths of cells that were treated with TRAIL in S/G2/M (n = 446) was compared to the control case. With a P-value of 0.54 [35], the null hypothesis of the two distributions originating from a distribution with the same median could not be rejected. (TIF) [file pcbi.1007812.s005.tif]

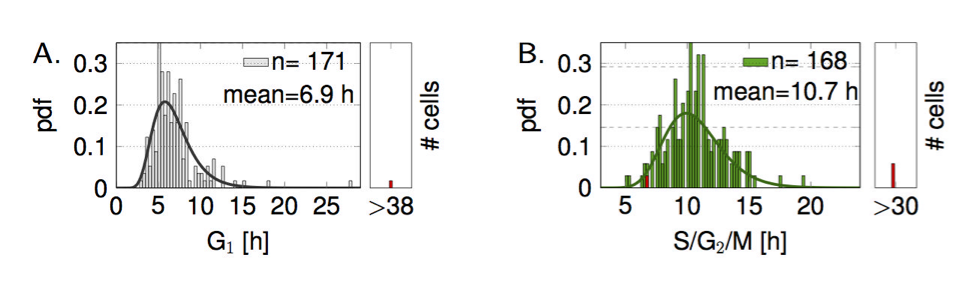

Supplement: S4 Fig — (A,B) Parameters of underlying lognormal distributions are μG1 = 1.87, σG1 = 0.34 and μSG2M = 2.34, σSG2M = 0.22 with mean values of 6.9 h and 10.7 h. Censored data (end of phase is not known) and outliers are highlighted in red. (TIF) [file pcbi.1007812.s006.tif]

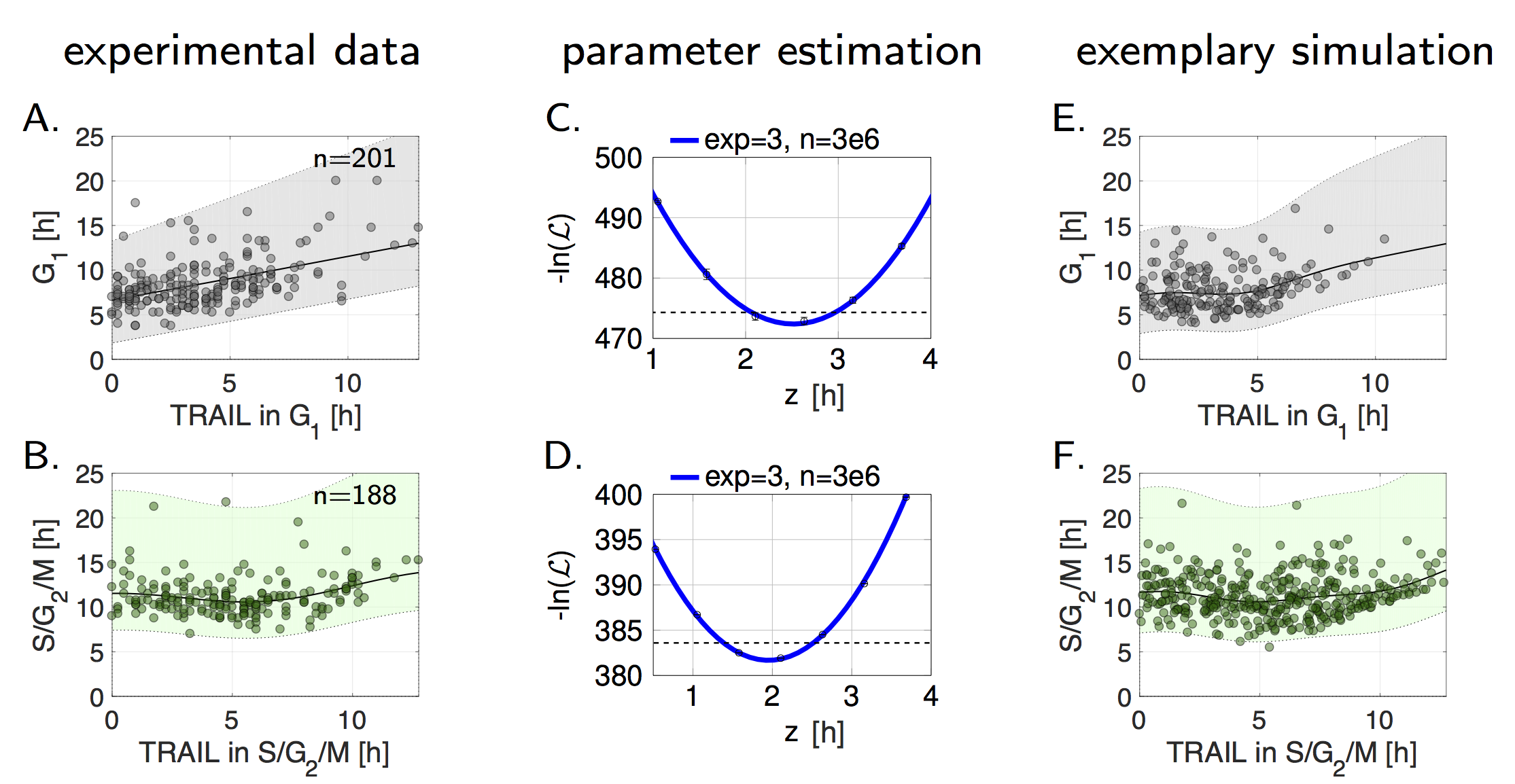

Supplement: S5 Fig — (A,B) Experimentally determined correlations of phase length and time of TRAIL addition. A linear gaussian process was assumed to highlight the 95% confidence interval. (C,D) Negative log-likelihood for varying prolongations of phases (z-values). 3 experiments with 3e6 samples were conducted. Mean and standard deviations are shown and a polynomial of 5th grade was fitted to resulting mean values. The lowest negative log-likelihood values were obtained for z^=2.5h (2.1 h–3 h), and z^=1.95h (1.4 h–2.5 h), respectively. Confidence bounds are indicated with dashed lines. (E,F) Exemplary model simulations with z^-values are presented. The 95% confidence intervals were calculated with a linear gaussian process, highlighted in gray and green. (TIF) [file pcbi.1007812.s007.tif]

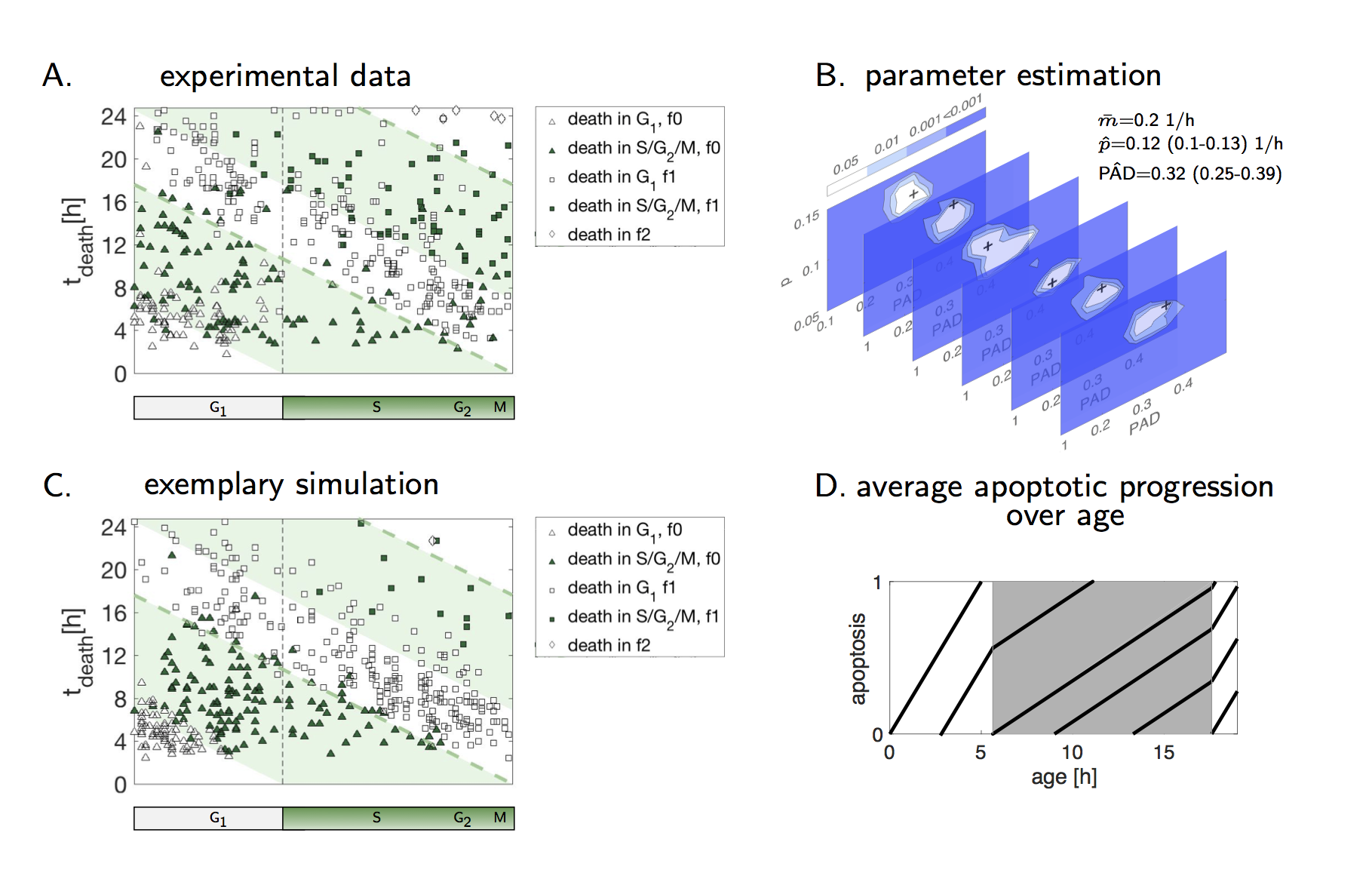

Supplement: S6 Fig — (A) Death times of experimental data (n = 538) were plotted against estimated initial cell cycle positions. Symbols indicate cell cycle phases at time of cell death. Experiment time was 24.5 h. Green shading highlights S/G2/M phase and the dashed line represents division of an averaged, untreated HCT-116/geminin cell. (B) Parameter estimation with six independent samples of initial conditions. Each sample size was approximately 1e4. m was estimated from cells dying in early G1. For simulation, the Matlab toolbox IQM Tools [46] was used and PAD was extended for the second generation so that p = 0 if Ci ∈ [0, PAD] or [1, PAD + 1] (see Eq 10). Significance levels are shown by color coding. Values of p^ and PAD^ represent mean and extreme values of six simulation experiments. (C) An exemplary simulation with best parameter values p^ and PAD^ is shown. (D) Average apoptotic progression in dependence on the ages of cells, representing the time from birth to TRAIL addition, is illustrated. The area of decelerated apoptosis progression is highlighted in gray. (TIF) [file pcbi.1007812.s008.tif]

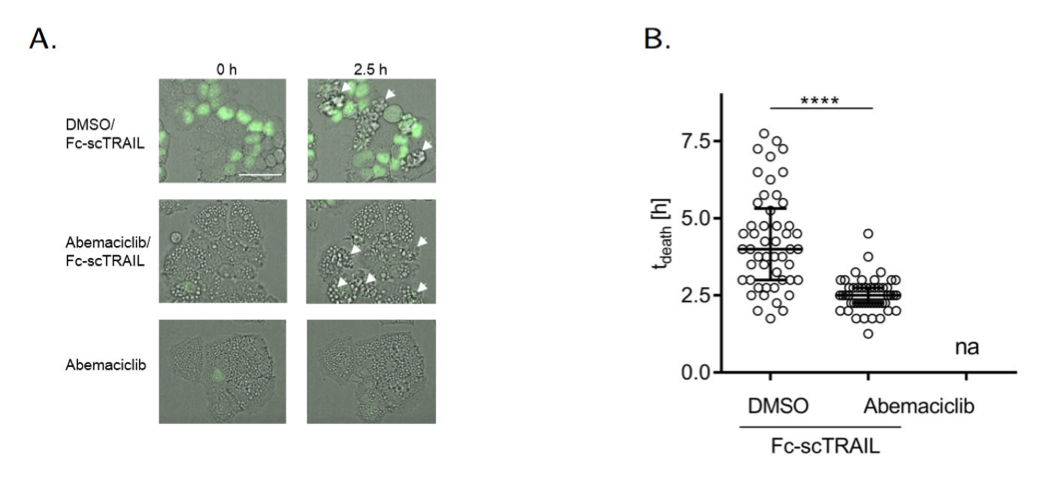

Supplement: S7 Fig — A. Representative time-lapse images of NCI-H460/geminin cells treated with Fc-scTRAIL (0.06 nM) or Abemaciclib (2 μM). Scale bar represents 50 μm. Dying cells are highlighted by white arrowheads. B. Times required to die (tdeath) of NCI-H460/geminin cells following treatment with Fc-scTRAIL alone or after pre-treatment with Abemaciclib (2 μM, 3 h). Shown are medians with interquartile ranges, plus min to max range from n = 50 cells observed in three independent experiments (**** p < 0.0001, unpaired t-test). Na = not applicable. (TIF) [file pcbi.1007812.s009.tif]
